# Impact of High-Pressure Process on Probiotics: Viability Kinetics and Evaluation of the Quality Characteristics of Probiotic Yoghurt

**DOI:** 10.3390/foods9030360

**Published:** 2020-03-19

**Authors:** Maria Tsevdou, Maria Ouli-Rousi, Christos Soukoulis, Petros Taoukis

**Affiliations:** 1 Laboratory of Food Chemistry and Technology, School of Chemical Engineering, National Technical University of Athens, 5 Heroon Polytechniou Str., 157 80 Athens, Greece; mtsevdou@chemeng.ntua.gr (M.T.); maria.ouli@gmail.com (M.O.-R.); 2 Luxembourg Institute of Science and Technology, Systems and Bioprocessing Engineering Group, Environmental Research and Innovation, 5 avenue des Hauts Fourneaux, L4362 Esch-sur-Alzette, Luxembourg; christos.soukoulis@list.lu

**Keywords:** probiotics, viability model, high-pressure processing, rheology, sensory quality, fermented dairy beverage

## Abstract

The impact of high-pressure (HP) processing on the viability of two probiotic microorganisms (*Bifidobacterium bifidum* and *Lactobacillus casei*) at varying pressure (100−400 MPa), temperature (20−40 °C) and pH (6.5 vs. 4.8) conditions was investigated. Appropriate mathematical models were developed to describe the kinetics of the probiotics viability loss under the implemented HP conditions, aiming to the development of a predictive tool used in the design of HP-processed yoghurt-like dairy products. The validation of these models was conducted in plain and sweet cherry-flavored probiotic dairy beverage products pressurized at 100−400M Pa at ambient temperature for 10 min. The microbiological, rheological, physicochemical and sensory characteristics of the HP-treated probiotic dairy beverages were determined in two-week time intervals and for an overall 28 days of storage. Results showed that the application of HP in the range of 200−300 MPa had minimal impact on the probiotic strains viability throughout the entire storage period. In addition, the aforementioned HP processing conditions enhanced the rheological and sensory properties without affecting post-acidification compared to the untreated product analogues.

## 1. Introduction

In recent decades, the functional food market has experienced a remarkable expansion as a result of the increasing consumer awareness for products that confer significant well-being and health promoting benefits. According to Food and Agriculture Organization/World Health Organization (FAO/WHO) definition, the term probiotics refers to “live microorganisms, which when administered in adequate amounts confer a health benefit to the host” [1]. In this context, only well-defined commensals and microbe consortia isolated from human samples with generic or core effects on gut physiology and supporting the health of reproductive tract, oral cavity, lungs, skin or brain-gut axis can be considered to be probiotics [2]. As for functional food innovation, fermented products such as yoghurt, cheese, fermented vegetables, fruit and legumes and dry cured meat are considered to be indigenous sources of probiotics [3]. The health benefits attained by the regular consumption of probiotic foods are associated with postbiotics, i.e., the production of secondary metabolites such as organic acids, enzymes, bioactive or antimicrobial peptides, exopolysaccharides, conjugated linoleic acids, vitamins, and phenolic compounds. Current knowledge of probiotics supports a plethora of therapeutic effects achieved following their regular administration to the human host, including their ability to relieve the symptomatology of irritable bowel syndrome, improve the blood serum lipid composition, stimulate the gut immunomodulation and prevent inflammation induced chronic disease such as obesity and several forms of cancer [4].

As far as the food industry practices, preserving the biological activity of probiotics is quite challenging as several endogenous, i.e., food matrix associated, and exogenous parameters, such as exposure of bacterial cells to harsh food processing, storage, and post-ingestion conditions, can potentially act as sublethal stressors of probiotic cells. For example, the availability of nutrients, bacterial cell growth promoters or inhibitors, the physical state (i.e., rubbery or glassy), the amount of dissolved oxygen, the pH and water activity conditions are among the commonest food matrix associated parameters that affect the viability of probiotics. In addition, food processing and storage can result in significant mechanical, heat, pH, osmolytic and pro-oxidants exposure induced injuries of the bacterial cells [5].

Despite their well-addressed biofunctional profile, fermented dairy products need to meet a handful of quality characteristics relating to texture, structure, olfactory, gustatory, and visual sensory modalities. In this context, the addition of food relevant structuring and texturizing agents, such as skim-milk powder, whey protein concentrates (WPCs), caseinates, polysaccharides, or other bulking agents, is considered to be a standard practice in dairy products manufacturing [6,7]. As an alternative, high-pressure (HP) processing has shown a great potential to deliver bespoke texture and structure reinforcing benefits to protein gels including fermented milk products [8,9,10,11,12,13]. It has been demonstrated that the implementation of a HP processing in a pressure-temperature range from 100 to 400 MPa and from 10 to 25 °C, respectively, and for an overall processing time of about 10−15 min, is sufficient to promote fermented milks structuring without the need for solid fortification. In addition, the aforementioned HP processing conditions can impart acceptable sensory cross-modality without affecting in an adverse way the biological activity of the living yoghurt starters and probiotic cells [14,15,16].

In the present work, it is hypothesized that the HP processing of pre-coagulated milk (stirred yoghurt) can lead to beneficial effects on its major physicochemical, rheological, and sensory properties without affecting significantly the viability of the embedding probiotic cells. For this purpose, the effect of HP processing conditions on the viability of two commonly used probiotic microorganisms (*Bifidobacterium bifidum* and *Lactobacillus casei*) in different pH values (pH 6.5 and 4.8) model systems was kinetically studied. The developed predictive models were assessed for their feasibility in the design of HP-processed real food systems, i.e., plain or cherry fruit-flavored probiotic yoghurts, containing *Bifidobacterium lactis* BB12 and *Lactobacillus acidophilus* LA5 strains. The probiotic yoghurts were evaluated concerning the microbiological, physicochemical, rheological, and sensory characteristics for an overall of 28 days of storage at chilling conditions.

## 2. Materials and Methods

### 2.1. Preparation of the Inocula for Kinetics Study

Stock cultures of the tested microorganisms (*Bifidobacterium bifidum* and *Lactobacillus casei*) were maintained in cryovials at −40 °C with glycerol (20% *v*/*v*) used as a cryoprotective agent. For the revival of each microorganism, one cryovial was transferred into 10 mL MRS broth (Merck, 1.10661, Darmstadt, Germany) and incubated at 37 °C for 24 h; for the revival of *Bifidobacterium bifidum*, 3% *v/v* of L-cysteine HCl sol. was added to MRS broth to achieve anaerobic environment. For growth and use in the kinetic experiments, 100 μL of the above inocula were transferred individually into 10 mL MRS broth (with 3% *v/v* of L-cysteine HCl sol. for *Bifidobacterium bifidum*) and incubated at 37 °C for 18−20 h. The final suspensions were transferred into 90 mL MRS broth with modified pH value to 4.80 (HCl sol. 1 M) or 6.50 (Na_2_HPO_4_/NaH_2_PO_4_ buffer sol. 0.1 M), representing the model system of low and high pH value respectively, and served as the inocula for viability loss experiments (microbial cells in stationary phase and initial plate counted at approximately 10^9^ CFU/mL). The selection of MRS growth medium instead of a milk-based medium was based on the fact that the viability of the tested microorganisms will not be affected by the possible presence of any protective agent (e.g., lipids) of the bacterial cells.

### 2.2. Yoghurt Preparation

Commercial homogenized pasteurized milk (3.2% *w*/*w* protein and 3.5% *w*/*w* fat) was placed into 2000 mL glass beakers and subjected to thermal treatment consisting of a rapid microwave assisted pre-heating step at 85 °C and a batch heating step at 85 °C for 20 min in a water-bath (Memmert, Guechenbach, Germany). Subsequently, milk was cooled to fermentation temperature (43.0±0.2 °C) and inoculated with 0.2% *v/v* commercial starter culture comprising *Streptococcus salivarius* subsp. *thermophilus* and *Lactobacillus delbrueckii* subsp. *bulgaricus* in a ratio of 4:1 (prepared as a 1:5 *w*/*w* dilution of Christian–Hansen freeze-dried YC-X11 culture in commercial UHT skim milk) and 0.3% *w*/*w* commercial probiotic cultures of *Bifidobacterium lactis* and *Lactobacillus acidophilus* (direct inoculation of Christian–Hansen BB12 and LA-5 probiotic cultures) according to the supplier’s instructions. The inoculated milk was placed in a water-bath maintained at the desirable fermentation temperature (43.0±0.4 °C) until the acidification end point (pH = 4.80) was achieved. Afterwards, the coagulum was manually stirred and divided in three batches. Two of the batches were single stage homogenized at 10 bar in a laboratory high-pressure homogenizer (APV1000, Kolding, Denmark) and packed in 180 g sterile multilayer pouches (laminate film: PP-aluminum-PE); one batch was stored at 5.0 ± 0.2 °C (**Control samples**), while the other was subjected to HP treatment (homogenized and HP-treated samples, coded as **Homo-HP**) and then stored under the same chilling conditions. A third batch prepared by directly packing the yoghurt in sterile multilayer pouches of 180 g for HP experiments (HP-treated samples, coded as **HP**) was also prepared. For cherry-flavored samples, 10% (*w*/*w*) of cherry syrup (Olympic Foods S.A., Athens, Greece) was blended with the yoghurt base until homogeneous dispersion of the syrup (68.6±1.57% *w/v* total sugars content of the syrup). The day after the production of the samples was considered to be the zero time point, while all quality characterization analysis were carried out in triplicate on a week time intervals for an overall of 28 days.

### 2.3. High-Pressure Processing 

The experiments were performed using a pilot-scale HP equipment (Food Pressure Unit FPU 1.01, Resato International BV, Assen, Netherlands), as previously described in Tsevdou et al. [12]. For kinetic experiments, 5 mL of the inocula were placed into pouches (laminate film: PP-aluminum-PE) and the viability loss experiments were conducted in the multi-vessel system, in duplicate at various combinations of pressure (100−400 MPa) and temperature (20−40 °C) for appropriate process times. For product batch experiments, 180 g of yoghurt were placed into multilayer pouches (laminate film: PP-aluminum-PE) and, the experiments were conducted in triplicate at various combinations of pressure (100−400 MPa) and ambient temperature (26.1±0.3 °C) for 10 min. 

### 2.4. Microbiological Analysis

Ten grams of yoghurt was transferred into a sterile stomacher bag with 90 g sterilized Ringer solution (1.15525, Merck, Darmstadt, Germany) and was homogenized for 60 s with a stomacher (BagMixer ^®^ Interscience, Saint-Nom-la-Bretèche, France). The enumeration of remaining viable cells of 10-fold serial dilutions of yoghurt homogenates was conducted using the appropriate plate methodology. 

For enumerating the probiotic bacteria, MRS agar (Merck, 1.10660, Darmstadt, Germany) was modified as follows; (a) addition of 5% *v/v* filter-sterilized NNLP (NNLP sol. comprises 100 mg Neomycin sulfate, 15 mg Nalidixic acid, 3 g Lithium chloride and 200 mg Paromomycin sulfate per 1 L (Tharmaraj & Shah, 2004)) sol. and 3% *v/v* filter-sterilized L-cysteine HCl sol. for Bifidobacteria spp., and (b) addition of 0.2% *v/v* filter-sterilized vancomycin hydrochloride sol. (Calbiochem, 627850, Darmstadt, Germany) for *Lactobacillus casei* enumeration [17]. Incubation of petri dishes was carried out in anaerobic jars (Merck, 1.16387, Darmstadt, Germany) at 37 °C for 72 h, using Anaerocult A (Merck, 1.13829, Darmstadt, Germany) as a catalyst. *Streptococcus thermophilus* was enumerated on M17 Agar (1.15108, Merck, Darmstadt, Germany) after incubation at 37 °C for 24 h under aerobic conditions. For *Lactobacillus bulgaricus* enumeration, the pour plate methodology on MRS agar with modified pH value at 4.58 was used, followed by incubation at 45 °C for 72 h in anaerobic jars as previously described. Two replicates of at least three appropriate dilutions were enumerated.

### 2.5. Physicochemical Analysis

The pH of yoghurt samples was measured using a pH meter (AMEL 338, Amel Instruments, Milano, Italy) whereas their titratable acidity (expressed as % lactic acid) was determined according to the International Dairy Federation (IDF) method [18].

The water holding capacity (WHC) of yoghurts was expressed as the grams of separated whey from 10 g of sample after centrifugation at 10.000 rpm (5.712 *g*) and 20 °C for 20 min [19].

The total color (E) of the samples were determined by measurement of CIELab values (L-value: lightness, a-value: redness/greenness, b-value: yellowness/blueness), using a CR200-Minolta Chromameter (Minolta Co., Chuo-Ku, Osaka, Japan) with an 8 mm measuring area, according to the equation:

(1) $$E=\sqrt{L^{2}+a^{2}+b^{2}}$$

The instrument was standardized using a white reference tile (Minolta Co., Chuo-Ku, Osaka, Japan). All measurements were carried out in triplicate.

### 2.6. Rheological Properties

The rheological behavior of yoghurt samples was determined using a rotational viscosimeter (RC1 Rheometer, Rheotec Meßtechnic GmbH, Raderburg, Germany) coupled with a circulating cooling bath (RE312, Lauda GmbH, Lauda-Königshofen, Germany). The measurements were carried out at 10 °C using a MS-CC48 DIN/FTK cylinder. 75 mL of yoghurt sample were transferred to the measuring cup and preconditioned at 10 °C for 1 h prior to analysis. Shear rate sweeps from 5 to 200 s^−1^ followed by constant shear rate step at 200 s^−1^ were applied. The duration of the shear stress sweep was 180 s. To describe the rheological behavior of the samples, the shear stress – shear rate data were fitted to the Ostwald–de Waale model:

(2) $$\sigma =K\cdot \gamma^{n}$$

where, *σ* is the shear stress (Pa·s), *γ* is the shear rate (s^−1^), *K* is the consistency index (Pa·s^n^) and *n* is the flow behavior index. The apparent viscosity (η, Pa·s) of the samples was calculated at a shear rate of 50 s^−1^, representing the sensing shear rate in the mouth of low viscosity foods [20]. 

### 2.7. Sensory Evaluation

Eight trained panelists (according to ISO standards) belonging to the staff of the Laboratory of Food Chemistry and Technology were recruited for assessing the plain and cherry-flavored yoghurts on days 1, 15 and 28 [21,22]. 

Two sessions per day (one in the morning and one in the afternoon) evaluating five formulations per session were conducted in the accredited according to ISO 17.025 Sensory Laboratory of NTUA (Athens, Greece) that has a standardized room (according to ISO standards) equipped with separate booths [23]. Samples were presented in plastic coded (three-digit random codes) cups, containing 50 mL of freshly removed from the refrigerator yoghurt samples. The panelists were asked to evaluate the samples using a 9-point intensity scale (1, lowest intensity; 9, highest intensity) based on pre-selected appearance (wheying-off, white color, yellowish color, cherry fruit color), tactile (ropy, uniform coagulum), orotactile (viscous, curdy, grainy), gustatory (sweet, bitter, sour, metallic) and olfactory (dairy flavor, sourmilk flavor, cherry flavor, rancid) sensory modalities. In addition, the panelists were asked to rate the yoghurt samples using a 9-point hedonic scale (1: I do not like at all, 9: I like extremely). During the sensory evaluation sessions, the panelists were instructed to cleanse their palate with low sodium spring water (Zagori, Greece) and consume a small piece of unsalted bread. The data were collected in specifically designed ballots and the panelists were encouraged to write down any criticisms on the tested products.

### 2.8. Data Analysis

First order kinetics was fitted to the logarithm of the concentration of viable cells [24]. The decimal reduction times (D, min) were estimated to describe the effect of process time on the viability loss of the tested microorganisms. The effect of temperature was expressed through the thermal resistance constant (z_T_), and the effect of pressure was described by the pressure resistance constant (z_P_) [24]. The parameters of the proposed mathematical model, which describes the viability loss of the microorganisms as a function of pressure and temperature, were estimated using non-linear regression on SYSTAT 10.0 software (SYSTAT 10.0 Statistics, 2002, SPSS Inc., Chicago, USA).

### 2.9. Statistical Analysis

The normal distribution of the data was verified by means of the Shapiro–Wilk test and Q-Q plot representation. In addition, the equality of variance within among the variables was verified using the Levene’s test. 3-factors ANOVA (treatment vs. pressure level vs. storage time) followed by Duncan’s means post hoc comparison test was applied for the analysis of all quality attributes in the shelf-life study of yoghurt samples. The physicochemical, rheological and sensory data matrices were log transformed and subject to Principal Components Analysis (PCA) to explore the qualitative affinities between the different products developed. All statistical analyses were performed using Statistica^®^ v.7 software (StatSoft Inc., Tulsa, OK, USA). Partial least squares regression (PLSR) using the leave-one-out validation method, as previously described in Kanta et al. [25], was used in order to explore the sensory modal drivers of degree of overall liking (DOL) of yoghurt samples. All PLS analyses were carried out using XLSTAT 2014 software (Addinsoft, UK).

## 3. Results and Discussion

### 3.1. Probiotics Viability Loss as a Function of Pressure and Temperature

The viability loss of *Bifidobacterium bifidum* as a function of pressurization time in model systems of different pH values at various combinations of pressure (100−400 MPa) and temperature (20−40 °C) was described by first order kinetics (R^2^ 0.84−0.99). The D-values were estimated at all studied pressure/temperature combinations (Table 1). The D-values decreased with increasing processing pressure and temperature at all levels tested, indicating the combined effect of temperature and pressure on the viability loss of these bacteria. In addition, *B. bifidum* exerted better survivability rates in high pH conditioned model systems, which corroborates previous studies reporting an optimal pH growth threshold in the range of 6.0−7.0 and a significant loss of survival for pH values below 4.5−5.0 [26,27].

At each temperature, the effect of pressure on the viability loss of *B. bifidum* was expressed through the pressure resistance constant (z_P_) (Table 1). When conducting the experiments in acid model system (4.80), the z_P_ value ranged from 84.7 MPa for processing at 20 °C to 98 MPa for processing at 35 °C (R^2^ range: 0.88−0.97), while in model system with pH value close to the optimum pH of growth (6.50), the z_P_ values ranged from 119 MPa when processing at 25 °C to 152 MPa when processing at 40 °C (R^2^ range: 0.89−0.99). Taking into account that in both laboratory and industrial scale HP equipment the minimum pressure setting step is 50 MPa, the z_P_ value for each model system was considered to be constant for the temperature range.

At each pressure, the effect of temperature on the viability loss of *B. bifidum* on the D-values was estimated using the Arrhenius equation and expressed in bacteriological terms by the thermal resistance constant (z_T_) (Table 1). All z_T_ values were estimated for all pressures tested (R^2^ range: 0.81–0.99), and it was observed that there is no specific trend when increasing the applied pressure, which is in accordance with findings in the literature for a variety of microorganisms [24,28].

**Figure 1:**
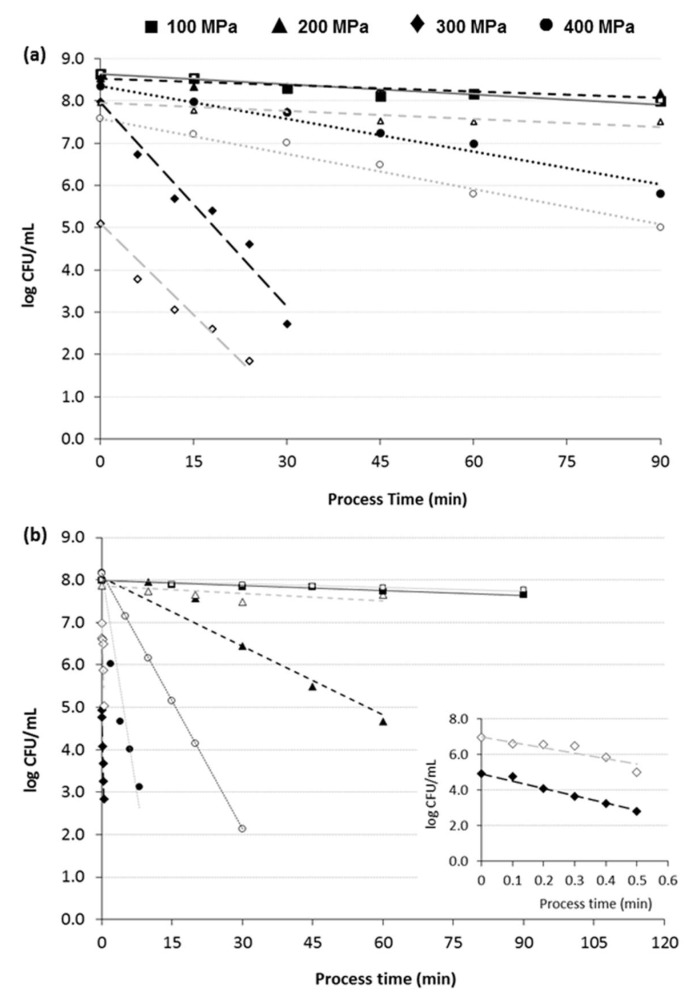
Comparison of *B. bifidum* (closed symbols-solid lines) and *L. casei* (open symbols-dashed lines) viability loss in broth model systems of pH value of (**a**) 6.5 and (**b**) 4.8, when pressurized at 30 °C (mean values ± standard deviation; linear lines represent the first order kinetics that describes the viability loss as a function of process time).

The viability loss of *Lactobacillus casei* was studied in growth media of both pH values. Results indicate that in growth medium with pH value of 6.50 (Figure 1a) *L. casei* exhibited similar viability loss behavior to that of *B. bifidum*, regardless the pressure applied. However, when *L. casei* was studied in the acid growth medium (Figure 1b), significant difference (*p* < 0.05) was observed in its viability as compared to that of *B. bifidum*, especially in the pressure range of 100−200 MPa. When high pressures were applied (300−400 MPa) both probiotic microorganisms exhibited similar viability loss. Similar results were obtained for all the pressure/temperatures combinations tested. 

The z_P_ value of *L. casei* was found similar of that of *B. bifidum* when tested in acid environment (91.7 ± 1.74 MPa in the temperature range of 20−35 °C, R^2^ range: 0.92−0.94), while when the experiments conducted in model system of high pH value (6.50), *L. casei* found to be slightly, yet significantly (*p* < 0.01), more baroresistant than the *B. bifidum* is (z_P_ value equal to 208 ± 6.53 MPa in the temperature range of 25−40 °C (R^2^ range: 0.95−0.96). There are no available data in the literature that could explain the observed baroresistance of Lactobacilli; however, several shelf-life studies in fermented dairy products have showed that both microorganisms lose their viability in pH values below 4.5−5.0, and that in higher pH values Lactobacilli are significantly less sensitive as compared to Bifidobacteria [29,30,31]. All z_T_ values of Lactobacilli were estimated for all pressures tested and, as in the case of *B. bifidum*, no specific trend was observed while increasing the applied pressure, with z_T_ values ranging from 15.8 to 45.1 °C and from 23.4 to 40.7 °C when the bacteria were studied in growth medium of pH 4.80 and 6.50, respectively.

### 3.2. Modeling Probiotics Viability Loss as a Function of Temperature and Pressure

A single multi-parameter model was developed to describe the effect of pressure and temperature process conditions only on the D-value of Bifidobacteria;

(3) $$D=D_{o}\cdot {\left(\exp\left\{-\frac{2.303\cdot T\cdot T_{ref}}{Z_{T}}\cdot \exp\left[-A\left(P-P_{ref}\right)\right]\cdot \left(\frac{1}{T}-\frac{1}{T_{ref}}\right)+\frac{2.303}{Z_{P}}\cdot \left(P-P_{ref}\right)\right\}\right)}^{-1}$$

where *D_0_* (min) is the decimal reduction time at the reference conditions of pressure (P_ref_, MPa) and temperature (T_ref_, °C), *A* is a constant parameter of the proposed model (MPa^−1^) and *z_T_* (°C) and *z_P_* (MPa) are the thermal and pressure resistance constants, respectively.

**Figure 2:**
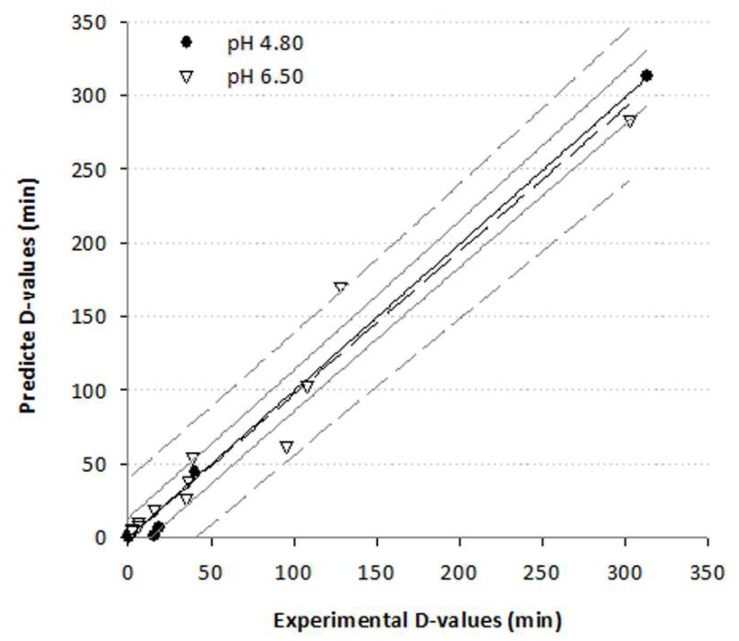
Correlation of experimental and predicted from the proposed model decimal reduction times (D) of *B. bifidum* tested in growth media of different pH values (Solid and dashed black lines represent the linear correlation of the data at pH of 4.8 and 6.5 respectively, while grey solid and dashed lines represent the corresponding 95% prediction bands).

This equation takes into account the effect of pressure on the z_T_ value, while the z_P_ value found not to be dependent on the process temperature. The parameters of the model were estimated (Table 2) using non-linear regression and, results indicated that the predicted D-values from the model were well correlated with the corresponding D-values obtained from the experimental data (Figure 2, R^2^ 0.97−0.99).

As detailed described above, considering that the survivability of *B. bifidum* was more sensitive to pressure and temperature conditions contrary to this of *L. casei*, we presumably deduce that the specific model can be a useful tool for estimating the survival of single or symbiotic bacterial cultures, comprising strains of the Bifidobacteria and Lactobacilli species, when exposed to HP processing conditions.

### 3.3. Selection of Optimal HP Conditions-Application in Yoghurt Production

Given that the application of HP processing at the pressure range of 100−300 MPa and temperature range of 20−25 °C for 10−15 min did not induce any significant lethality to the probiotic cells population under acidic conditions, we decided to assess the feasibility of these processing conditions when applied to a fermented probiotic dairy food. Treatment of yoghurt samples was also performed at 400 MPa, to explore possible positive effect on the rheological parameters at this higher pressure. The physicochemical, rheological and sensory properties as well as the probiotics cells survivability over a 28 days storage period were monitored.

#### 3.3.1. Viability of Starter Culture and Probiotic Bacteria in Yoghurt Samples

The total viable counts of the starter culture (*Str. thermophilus* and *L. bulgaricus*) in the plain probiotic yoghurt samples after HP treatment and during storage are given in Table 3.

HP processing of the probiotic yoghurts induced a reduction of the starter culture total load accounting for ca. 0.2−0.5 log_10_ CFU/g for HP-treated products and ca. 0.2−0.3 log_10_CFU/g for Homo-HP-treated products. Throughout storage, starter culture cells underwent a pressure dependent decrease in their viability of about 0.2−0.5 log_10_CFU/g; yet their total counts were well above the recommended level of 7.0 log_10_ CFU/g [32]. When probiotic yoghurts were high pressurized at 400 MPa, (in the absence or presence of conventional homogenization step), a greater decrease in starter culture total counts was observed which were well below the WHO/FAO acceptable thresholds.

**Figure 3:**
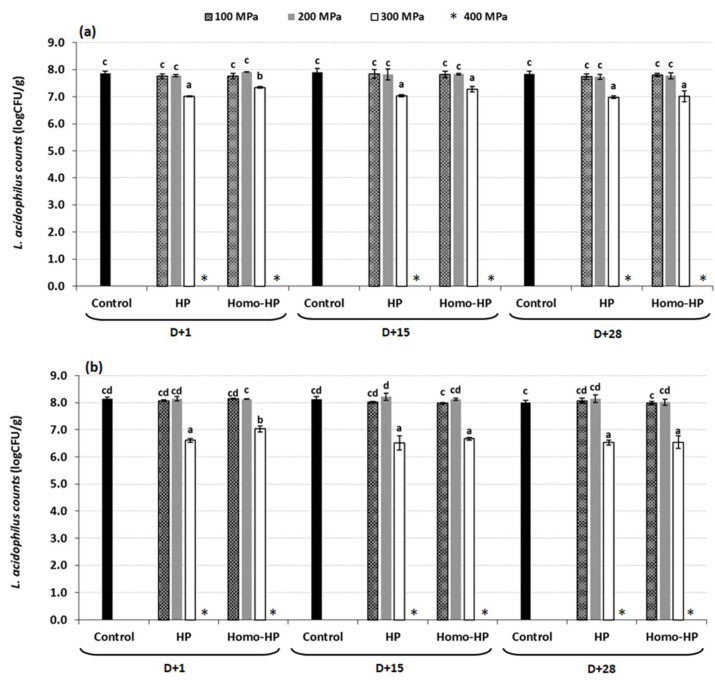
Viability of *Lactobacillus acidophilus* LA5 during 28 days of storage at 5°C in different treated (**a**) plain and, (**b**) cherry-flavored probiotic yoghurt (Letter D indicates the day of production. Different letters among bars indicate significant difference (*p* < 0.05) between tested samples according to Duncan’s mean values post hoc comparison test.).

The remaining probiotic counts of probiotic dairy beverages after HP treatment and during storage are shown in Table 4 for *Bifidobacterium lactis* BB12 and in Figure 3 for *Lactobacillus acidophilus* LA5.

In agreement with the probiotics survival findings in the model system, pressure increase resulted in significantly higher sub-lethality of Bifidobacteria cells compared to Lactobacilli. Bifidobacteria cell population underwent a decrease of about 0.1 to 1.4 log_10_ CFU/g or 0.1 to 1.1 log_10_ CFU/g for HP and Homo-HP-treated samples, respectively, when the products treated in the pressure range of 100−300 MPa. The corresponding decrease in Lactobacilli population was 0.1−0.9 log_10_ CFU/g and 0.0−0.6 log_10_ CFU/g for HP and Homo-HP-treated samples, respectively. Similar results for probiotics viability were obtained during storage, where a further slight decrease in probiotic populations of ca. 0.2−0.7 log_10_ CFU/g were observed for all tested samples. Based on these observations it can be hypothesized that after HP treatment there are potentially more injured cells unable to recover in the case of Bifidobacteria than of Lactobacilli. Despite the observed decrease, when products were pressurized in the range of 100−300 MPa, the remaining probiotic population was above the recommended level of 10 ^6^ CFU/g for both probiotic strains and both treated products (HP and Homo-HP samples) [1]. In the HP-treated products the decrease in probiotic cells was greater than the viability loss observed for Homo-HP-treated products. However, during storage at 5 °C for 28 days, Homo-HP samples presented higher viability loss of probiotic cultures as compared to that of HP samples. Similar to the case of starter culture microorganisms, when products were pressurized at 400 MPa, counts of both probiotic strains decreased below the legislative acceptable levels in all alternatively treated products (depicted as N.D. in Table 4 and, * symbol in Figure 3). 

In the case of cherry-flavored yoghurt samples, a similar reduction of about 0.2−0.4 log_10_ CFU/g was observed for total starter culture population when yoghurt samples were subjected to HP processing (both HP and Homo-HP samples), followed by a further decrease of 0.2−0.4 log_10_ CFU/g during 28 days of storage. Probiotic populations in cherry-flavored yoghurt samples subjected to HP processing underwent a reduction of approximately 0.2−1.8 log_10_ CFU/g, followed by a further decrease of 0.2 and 0.1−0.5 log_10_ CFU/g for BB12 and LA5 populations, respectively, during 28 days of storage, indicating a significant lower reduction at the first day of production and during storage to that observed for plain yoghurt samples. Addition of syrup resulted in an increase in the amount of polysaccharides and sugars in these samples, a growth factor for probiotics survival, indicating that the use of substances for the enhancement of taste and flavor in dairy products could also improve the viability of microbial populations [33].

#### 3.3.2. Physicochemical/Rheological Characteristics After HP Treatment and During Storage

**Figure 4:**
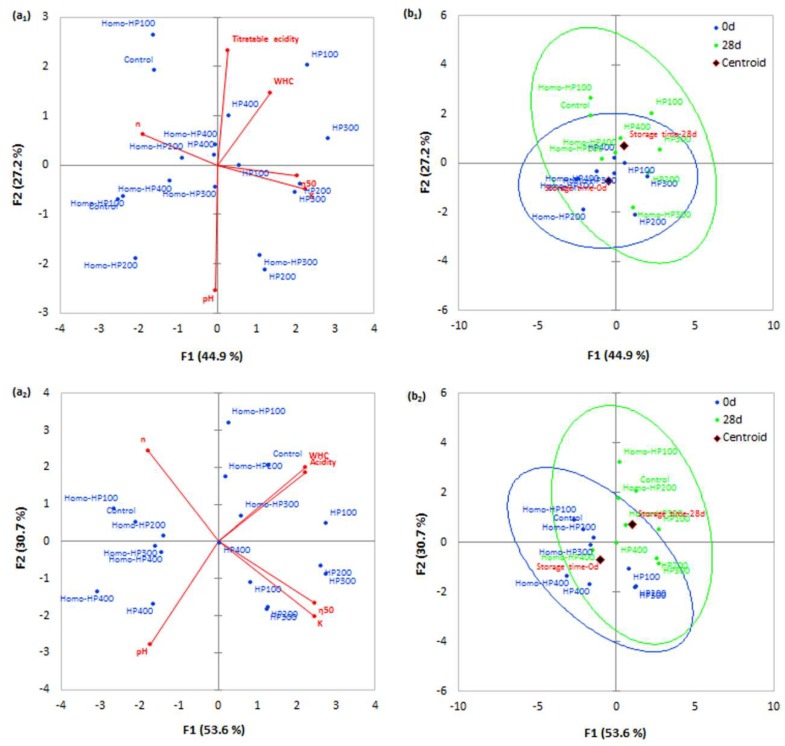
Principal components analysis (a_1_,b_1_: loadings plot, a_2_,b_2_: scores plot) for the classification of plain (**a**) and cherry-flavored (**b**) yoghurt samples stored at 5 °C for 28 d, based on their physicochemical characteristics (principal components 1 and 2 accounted together the 72.1% and 84.3% of the total variance explained for plain and cherry-flavored yoghurts, respectively).

The physicochemical (pH, acidity, and WHC) and rheological parameter (consistency coefficient, rheological behavior index and apparent viscosity) data matrices for plain and cherry-flavored yoghurts were averaged, standardized and subjected into PCA (Figure 4).

As seen in Figure 4, the titratable acidity was reduced proportionally to the intensity of the pressurization process for both plain and cherry-flavored products, which implies that the metabolic activity of the probiotic microorganisms and yoghurt starter is significantly slowed down [34,35]. The post-acidification was found to be pronouncedly higher in the case of cherry-flavored formulations for the entity of the tested processing conditions (HP and Homo-HP) and thus, it is assumed that the presence of readily available nutrient sources in the fruit syrup (e.g., sugar, glucose syrup, natural presence of sugars and fibers in cherry juice) stimulated the growth of the microbiota throughout the chilling storage. 

Expectedly the cherry syrup addition suppressed the total color index from 84.5 to 78 for the control systems. A similar trend regarding the impact of the HP processing on total color i.e., from 79.6−85.9 to 75.1−78.9, was observed. However, no significant differences between the HP and Homo-HP samples were identified. Interestingly, the increase in the intensity of pressurization process resulted in a reduction of the total color of cherry-flavored yoghurts, most probably due to the degradation or isomerization of naturally occurring pigmenting compounds such as anthocyanins and carotenoids [36,37]. 

The WHC is a measure of the ability of acid gel to retain unbound water (loosely hold in the interspaces of the protein gel network) on the application of mechanical stress. It has been demonstrated that the more uniform association between denatured beta-lactoglobulin (as it is more sensitive than alpha-lactalbumin to high-pressure treatment) and the dissociation and re-aggregation of the micelle fragments occurring during the HP processing reduce the proneness of the acid protein gels to syneresis [13]. According to our findings, the WHC in the HP-treated plain yoghurts was significantly higher than that of the Homo-HP-treated ones. On the other hand, a reversed behavior was observed in the case of cherry-flavored yoghurts, where the Homo-HP exerted the highest WHC values. Although there is no conclusive explanation, it is presumed that the breakdown of the acid gel during the homogenization step, allowed the stabilizing agents (e.g., pectins) found in the syrup base, to occupy a higher hydrodynamic volume due to the reduction of steric hindrances, and thus it improved the ability of the overall acid protein/stabilizer protein network to retain more water via hydrogen bond bridging.

The implementation of the HP treatment at the final stage of the production process appeared to improve the rheological properties of the end product, either when it is applied individually or subsequently to conventional homogenization. 

The values of consistency coefficient (*K*) of plain yoghurt beverages were significantly (*p* < 0.001) affected by the applied treatment, the level of the applied pressure and storage time. HP treatment of samples in a pressure range of 100−300 MPa led to an increase in consistency coefficient values compared to the controls (homogenized at 10 bar after the break down of the coagulum). At 400 MPa, consistency coefficient values decreased, although it was still higher than control samples. Flow behavior index values of plain yoghurt beverages were also significantly (*p* < 0.001) affected by the applied treatment and storage time, showing a significant decrease with increasing HP, either applied individually (HP samples) or subsequently to conventional homogenization (Homo-HP samples). This trend was reversed at 400 MPa. The apparent viscosity of plain yoghurt samples increased by 162, 188, 190 and 68% at pressures of 100, 200, 300 and 400 MPa, and 26, 46, 65 and 29% when HP was applied subsequently to conventional homogenization at the same pressures. 

The degree of whey protein denaturation is a very important factor that affects the rheological behavior of the coagulum and can be related to the intensity of the pressure applied. It is also responsible for the protein-protein interactions, and the retention of whey proteins in the network gel. HP processing lead to enhanced whey protein hydrophobicity, resulting in an increase in the binding affinity of whey proteins and thus, alterations on their structure and improvement of their functional properties [38,39]. Moreover, it has been reported that when the applied pressure exceeds 200 MPa, a partial disintegration of casein micelles occurs, which results in an increased number of fragments of casein solution and in greater solvation of the protein. The partial fragmentation of casein micelles is accompanied by the solubilization of colloidal calcium phosphate, suggesting that the structure of the gels obtained is dominated by casein-casein interactions instead of the interactions of whey protein-casein, forming small particles that are often shaped into clumps and chains, and therefore into compact and strengthened protein networks [11]. The increase in yoghurt viscosity, with pressure, have been previously related to modifications of beta-lactoglobulin structure, since significant molecular unfolding and further protein aggregation occurs, especially when pressures above 100 MPa are applied and with a threshold at 400 MPa, where usually the coagulum becomes more coarse and an increase in syneresis is observed [16].

The parameters of consistency coefficient and flow behavior index of cherry-flavored yoghurt beverages were affected by the applied treatment similarly to plain yoghurt beverages. However, storage time did not seem to significantly affect these parameters, probably due to the increase in total solids and the presence of small amounts of pectin in the syrup that could have stabilized the structure of the coagulum.

#### 3.3.3. Sensory Profile of the HP Yoghurts

**Figure 5:**
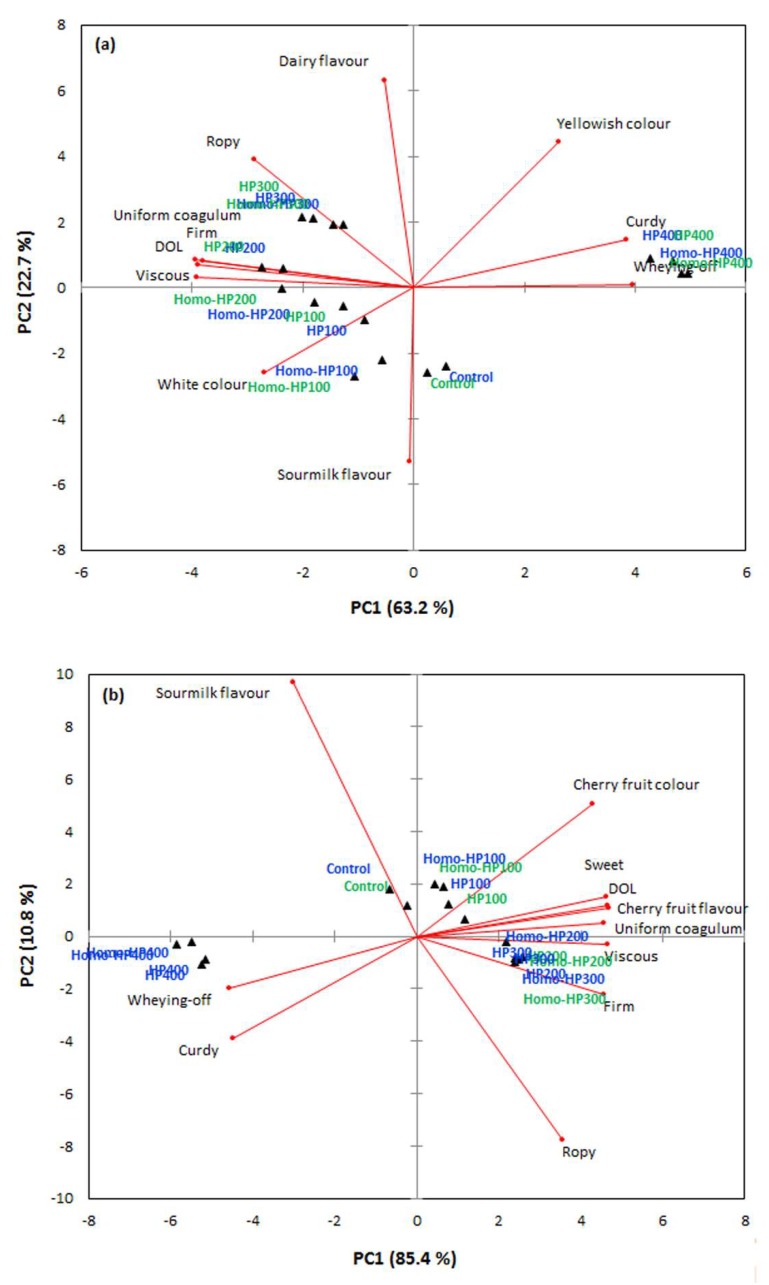
Principal components analysis (**a**) plain yoghurt, (**b**) cherry-flavored yoghurt for the classification of the yoghurt samples at 0d (blue) and 28d (green) based on their sensory characteristics (principal components 1 and 2 accounted together the 96.2% of the total variance explained).

To understand the impact of the processing conditions on the sensory profile of the plain and cherry fruit-flavored yoghurt products, the sensory modalities score data at the beginning and end of the storage time were subjected to PCA (Figure 5). Prior to analyses, the sensory modalities that were not significantly (*p* > 0.05) influenced by the independent processing factors were excluded from the PCA analysis. In this context, one orotactile (grainy), two gustatory (bitter and metallic) and one olfactory (rancid flavor) modality were not affected by processing conditions in both plain and cherry-flavored products. Moreover, white color was not assessed in the case of cherry-flavored yoghurts and accordingly, cherry flavor and cherry fruit color were excluded from the sensory lexicon used in the evaluation of plain yoghurts. 

As seen in Figure 5a, the HP processing of the plain yoghurts was primarily associated with the modification of their tactile and oro-tactile aspects. Plain yoghurts pressurized at 100 to 300 MPa exhibited rather a textural and structural affinity in terms of ropiness, coagulum uniformity, thickness, and firmness. Although the increase in the pressure intensity appeared to intensify the aforementioned sense stimuli, the differences between the samples pressurized at 200 and 300 MPa were not significant. On the other hand, further increase in the pressure, i.e., 400 MPa, resulted in colloidally non-uniform, curdy-like products that were prone to gel structural collapse as indicated by the significant evidence of syneresis. As far as concerns the assessed flavor—taste attributes, the increase in the HP process intensity was accompanied by the increase in dairy and partial masking of sourmilk flavor modalities. The observed reverse correlation between dairy/milky and sour/sourmilk or even astringent sense stimuli has been reported in several studied on acidified dairy products [40,41]. Although the amount of organic acids, i.e., lactic, formic or orotic acid, are considered to be the major drivers of the sour-like flavor and taste modalities, the acid protein gel structural conformation can also have a significant role on the partitioning of flavor volatile compounds that contribute to the development of fermented milk olfactory modalities such as acetaldehyde, acetoin, diacetyl, acetone etc. [42,43]. Thus, the HP induced enhancement of the structural integrity of the acid gels can reduce the partitioning coefficients of the aforementioned flavor compounds leading to a more dairy/buttery-like flavor profile. 

In the case of the cherry-flavored yoghurts (Figure 5b), the addition of the fruit syrup did not modified remarkably the interplay between the sensory cross-modal perception and the intensity of the HP processing step. As with plain yoghurts, cherry-flavored yoghurts processed at 200−300 MPa exerted the highest intensities of thickness, ropiness, coagulum uniformity and gel firmness and at the same time received the highest scores for cherry fruit flavor and sweet taste modalities. The detrimental effects of the excessive pressure processing on the structural integrity of the coagulum were also observed in the case of the cherry fruit-flavored products.

**Figure 6:**
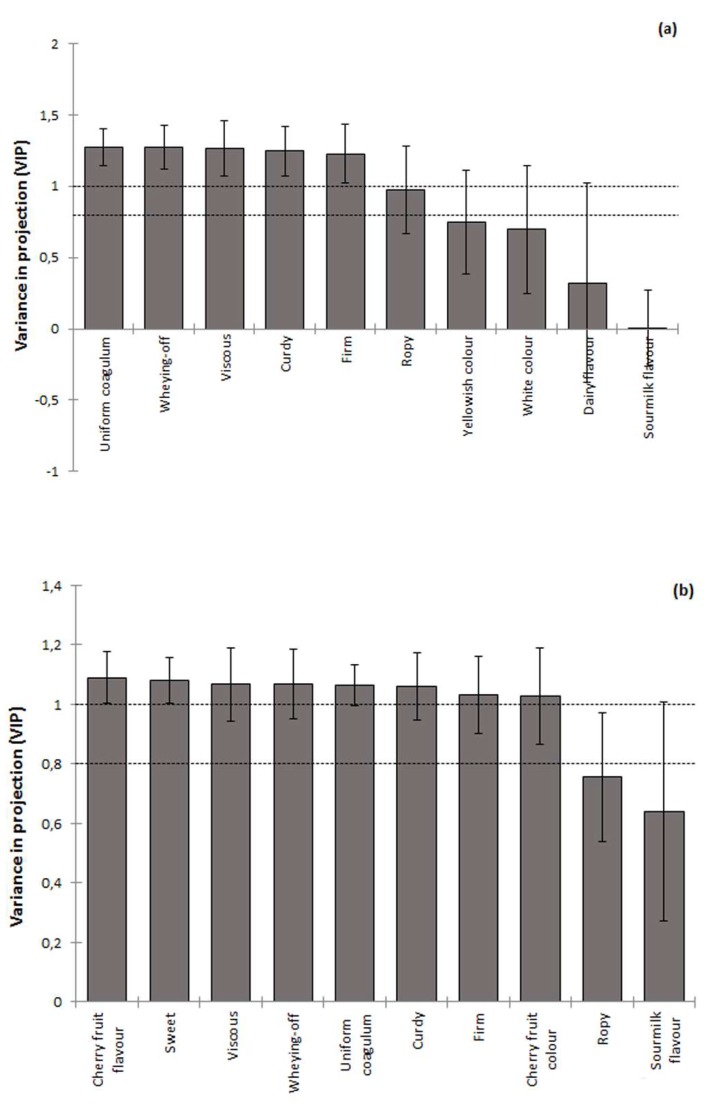
Variable in Project values (VIP) in descending order as calculated according to the partial least squares regression (PLSR) analysis. (**a**) VIP calculated for plain yoghurts according to PLSR analysis; (**b**) VIP calculated for cherry fruit-flavored yoghurts according to PLSR analysis.

With regards to the hedonic assessment of the products, the PCA analysis suggested that the DOL is dependent of the product formulation. To further explore the sensory modal drivers of DOL, the standardized and averaged dataset were subjected to PLSR using the leave-one-out validation. As seen in Figure 6a for plain yoghurts, the DOL was mainly driven by tactile/orotactile properties (VIP > 1) and therefore, the structural and colloidal integrity of the formed acid gels has the most important role for the acceptability of the final products. On the other hand, the drivers of overall liking of cherry fruit-flavored yoghurts appeared to be more complex, as not only the texture relating but also the fruit flavor and color attributes classified as impacting attributes (Figure 6b). Finally, the storage time had only minor (*p* > 0.05) effects on the sensorial quality and overall liking of both type of yoghurts, most probably due to the very mild post-acidification and colloidal change phenomena occurring throughout the tested storage period.

## 4. Conclusions

The recommended HP conditions in the literature (200 MPa at 20−25 °C for process time for up to 10−15 min) for texture improvement in dairy products was found not detrimental to the viability of the examined probiotic bacteria. In the case of model systems, the maximum calculated inactivation due to HP process was about 0.4 log_10_ CFU/mL. To describe the effect of pressure and temperature process conditions on the viability of the tested probiotic bacteria, a single multi-parameter equation was proposed, and the parameters of this model were estimated for the most baro-sensitive probiotic bacterium (i.e., *Bifibobacterium bifidum*). When HP processing was applied in probiotic yoghurt at the final stage of production, especially when treated at 200−300 MPa, the quality and sensorial properties of the final product were improved. The viscosity of the final product increased and the whey separation decreased, while the viability loss of the probiotic microorganisms ranged between 0.5−1.2 log_10_ CFU/g, and no significant viability loss was observed during refrigerated storage for 28 days. Overall, HP process can be successfully applied in such dairy products at the final step of production in order to improve their quality attributes and extend their shelf life without the need for stabilizer addition, while acceptably affecting their functionality. Moreover, the addition of flavor enhancement substances seems to improve the rheological properties of the coagulum but also increase the viability of the probiotic bacteria. Further research could focus on the effect of the addition of prebiotics in similar treated dairy products both on probiotics viability and their quality indices.

## Figures and Tables

**Table 1:** Viability loss kinetic parameters of *Bifidobacterium bifidum.*

**Decimal Reduction Times (D, min) of *B. bifidum* in pH 4.80**					
	**20 °** **C**	**25 °** **C**	**30 °** **C**	**35 °** **C**	**z_T_ (°** **C)**
**100 MPa**	435 (±23)	400 (± 19)	263 (± 5.2)	250 (± 7.3)	**62.2 (± 16)**
**200 MPa**	313 (±9.0)	40.3 (± 1.4)	18.6 (± 2.0)	16.2 (± 0.7)	**11.9 (± 3.2)**
**300 MPa**	4.08 (±0.01)	2.53 (± 0.04)	1.43 (± 0.1)	0.71 (± 0.06)	**19.8 (± 1.7)**
**400 MPa**	0.25 (±0.03)	0.27 (± 0.01)	0.24 (± 0.01)	0.11 (± 0.00)	**37.5 (± 3.4)**
**z_P_ (MPa)**	**90.9 (± 5.1)**				
**Decimal Reduction Times (D, min) of *B. bifidum* in pH 6.50**					
	**25 °C**	**30 °C**	**35 °C**	**40 °C**	z_**T**_** (°C)**
**100 MPa**	-	526 (± 15)	333 (± 2.1)	185 (± 9.0)	22.1 (± 2.3)
**200 MPa**	303 (± 3.0)	128 (± 10)	108 (± 9.3)	95.2 (± 7.0)	78.4 (± 9.4)
**300 MPa**	38.6 (± 2.1)	36.5 (± 1.9)	35.2 (± 0.6)	15.8 (± 2.0)	39.7 (± 1.6)
**400 MPa**	6.47 (± 1.1)	6.20 (± 0.9)	2.22 (± 0.3)	2.09 (± 0.4)	28.9 (± 1.2)
**z_P_ (MPa)**	**151 (± 8.0)**				

Values are means ± standard error of regression.

**Table 2:** Viability loss kinetic parameters of *Bifidobacterium bifidum.*

Parameter	Estimated Value pH 4.80	Estimated Value pH 6.50
P_ref_ (MPa)	200	200
T_ref_ (°C)	25	25
D_o_ (min)	44.5 ± 6.39	281 ± 19.7
z_T_ (°C)	5.91 ± 0.44	22.9 ± 3.50
z_P_ (MPa)	90 (constant)	140 (constant)
A (MPa^−1^)	0.016 ± 0.002	0.002 ± 0.000
**R^2^**	**0.99**	**0.95**

Values are means ± standard error of regression.

**Table 3:** Viability of starter culture (as total numbers of *Str. thermophilus* and *L. bulgaricus*) in different treated plain and cherry-flavored yoghurt during storage.

Plain Yoghurt				
Control *	-			
D + 1	8.9 ± 0.2 ^i^	-	-	
D + 15	8.8 ± 0.1 ^hi^			
D + 28	8.7 ± 0.0 ^gh^			
**HP**	**100 MPa**	**200 MPa**	**300 MPa**	**400 MPa**
D + 1	8.7 ± 0.1 ^fgh^	8.7 ± 0.1 ^fgh^	8.4 ± 0.2 ^bcd^	N.D.
D + 15	8.5 ± 0.1 ^defg^	8.2 ± 0.3 ^abc^	8.5 ± 0.1 ^def^	N.D.
D + 28	8.5 ± 0.1 ^def^	8.2 ± 0.1 ^ab^	8.5 ± 0.1 ^def^	N.D.
**Homo-HP**	**100 MPa**	**200 MPa**	**300 MPa**	**400 MPa**
D + 1	8.7 ± 0.2 ^gh^	8.6 ± 0.1 ^efgh^	8.6 ± 0.0 ^defgh^	N.D.
D + 15	8.5 ± 0.0 ^defgh^	8.1 ± 0.1 ^a^	8.6 ± 0.0 ^defgh^	N.D.
D + 28	8.4 ± 0.0 ^cde^	8.1 ± 0.1 ^a^	8.5 ± 0.1 ^defg^	N.D.
**Cherry-Flavored Yoghurt**		**-**	**-**	**-**
Control *	**-**	**-**	**-**	**-**
D + 1	8.8 ± 0.0 ^ij^	-	-	-
D + 15	8.4 ± 0.0 ^bcd^			
D + 28	8.3 ± 0.1 ^bc^			
**HP**	**100 MPa**	**200 MPa**	**300 MPa**	**400 MPa**
D + 1	8.9 ± 0.0 ^j^	8.6 ± 0.0 ^fgh^	8.5 ± 0.1 ^cde^	N.D.
D + 15	8.8 ± 0.1 ^ij^	8.2 ± 0.1 ^a^	8.3 ± 0.1 ^b^	N.D.
D + 28	8.5 ± 0.0 ^def^	8.3 ± 0.1 ^bc^	8.5 ± 0.1 ^cde^	N.D.
**Homo-HP**	**100 MPa**	**200 MPa**	**300 MPa**	**400 MPa**
D + 1	8.7 ± 0.1 ^hi^	8.7 ± 0.1 ^hi^	8.6 ± 0.1 ^fg^	N.D.
D + 15	8.6 ± 0.0 ^gh^	8.5 ± 0.0 ^cde^	8.4 ± 0.0 ^bc^	N.D.
D + 28	8.5 ± 0.1 ^efg^	8.3 ± 0.0 ^b^	8.2 ± 0.0 ^a^	N.D.

* Control samples were not HP-treated but only homogenized at 10 bar after the break of the coagulum. Letter D indicates the day of production. N.D. indicates microbial load below the acceptable limit of 7.0 log_10_ CFU/g of the total microbial counts for the starter culture at the end of shelf life. Different letters indicate significant difference (*p* < 0.05) between tested samples according to Duncan’s mean values post hoc comparison test.

**Table 4:** Viability of *Bifidobacterium lactis* BB12 in different treated plain and cherry-flavored yoghurt during storage.

**Plain Yoghurt**				
Control *	**-**			
D + 1	8.2 ± 0.1 ^jk^	-	-	
D + 15	8.3 ± 0.2 ^jk^			
D + 28	8.3 ± 0.1 ^k^			
**HP**	**100 MPa**	**200 MPa**	**300 MPa**	**400 MPa**
D + 1	8.1 ± 0.1 ^hijk^	7.9 ± 0.0 ^fghi^	6.8 ± 0.3 ^c^	N.D.
D + 15	8.1 ± 0.1 ^ghijk^	7.8 ± 0.1 ^fg^	6.3 ± 0.1 ^ab^	N.D.
D + 28	8.1 ± 0.1 ^ghijk^	7.8 ± 0.1 ^fgh^	6.1 ± 0.1 ^a^	N.D.
**Homo-HP**	**100 MPa**	**200 MPa**	**300 MPa**	**400 MPa**
D + 1	8.1 ± 0.1 ^fghij^	8.0 ± 0.1 ^fghij^	7.1 ± 0.2 ^d^	N.D.
D + 15	8.1 ± 0.0 ^ijk^	7.8 ± 0.3 ^f^	6.7 ± 0.1 ^c^	N.D.
D + 28	8.1 ± 0.1 ^hijk^	7.4 ± 0.1 ^e^	6.5 ± 0.1 ^bc^	N.D.
**Cherry-Flavored Yoghurt**		**-**	**-**	**-**
Control *	**-**	**-**	**-**	**-**
D + 1	8.4 ± 0.0 ^e^	-	-	-
D + 15	8.2 ± 0.0 ^d^			
D + 28	8.1 ± 0.1 ^d^			
**HP**	**100 MPa**	**200 MPa**	**300 MPa**	**400 MPa**
D + 1	8.2 ± 0.0 ^d^	7.9 ± 0.0 ^c^	6.5 ± 0.1 ^a^	N.D.
D + 15	8.1 ± 0.0 ^d^	7.7 ± 0.0 ^bc^	6.5 ± 0.1 ^a^	N.D.
D + 28	8.1 ± 0.1 ^d^	7.7 ± 0.1 ^bc^	6.5 ± 0.2 ^a^	N.D.
**Homo-HP**	**100 MPa**	**200 MPa**	**300 MPa**	**400 MPa**
D + 1	8.2 ± 0.1 ^d^	7.8 ± 0.0 ^c^	6.6 ± 0.2 ^a^	N.D.
D + 15	8.2 ± 0.0 ^d^	7.8 ± 0.0 ^c^	6.4 ± 0.2 ^a^	N.D.
D + 28	8.1 ± 0.1 ^d^	7.6 ± 0.1 ^b^	6.4 ± 0.1 ^a^	N.D.

* Control samples were not HP-treated but only homogenized at 10 bar after the break of the coagulum. Letter D indicates the day of production. N.D. indicates microbial load below the acceptable limit of 7.0 log_10_ CFU/g of the total microbial counts for the starter culture at the end of shelf life. Different letters indicate significant difference (*p* < 0.05) between tested samples according to Duncan’s mean values post hoc comparison test.
